# Supplementary material for: A population-based serological study of post-COVID syndrome prevalence and risk factors in children and adolescents
Source: Nat Commun. 2022 Nov 29;13:7086. doi: 10.1038/s41467-022-34616-8 (PMC9708639; doi:10.1038/s41467-022-34616-8)
Supplement: Supplementary file 1 — Supplementary Information [file 41467_2022_34616_MOESM1_ESM.pdf]

A population-based serological study of post-COVID syndrome prevalence and risk factors in children and adolescents

Roxane Dumont[1], Viviane Richard[1], Elsa Lorthe[1], Andrea Loizeau[1], Francesco Pennacchio[1], María-Eugenia Zaballa[1], Hélène Baysson[1,2], Mayssam Nehme[2], Anne Perrin[3], Arnaud G. L'Huillier[3,4], Laurent Kaiser[5,6,7], Rémy P. Barbe[8], Klara M. Posfay-Barbe[3,4], Silvia Stringhini[1,2,9*], SEROCoV-KIDS study group & Idris Guessous[2,10*].

1 Unit of Population Epidemiology, Division of Primary Care Medicine, Geneva University Hospitals, Geneva, Switzerland
2 Department of Health and Community Medicine, Faculty of Medicine, University of Geneva, Geneva, Switzerland
3 Division of General Pediatrics, Department of Woman, Child, and Adolescent Medicine, Geneva University Hospitals, Geneva, Switzerland
4 Pediatric Infectious Diseases Specialist, Geneva University Hospitals and Faculty of Medicine, Geneva, Switerland
5 Geneva Center for Emerging Viral Diseases and Laboratory Virology, Geneva University Hospitals, Geneva, Switzerland.
6 Division of Laboratory Medicine, Geneva University Hospitals, Geneva, Switzerland
7 Department of Medicine, Faculty of Medicine, University of Geneva, Geneva, Switzerland
8 Division of Child and Adolescent Psychiatry, Department of Woman, Child, and Adolescent Medicine, Geneva University Hospitals, Geneva, Switzerland
9 University Center for General Medicine and Public Health, University of Lausanne, Lausanne, Switzerland
10 Division and Department of Primary Care Medicine, Geneva University Hospitals, Geneva, Switzerland
*These authors contributed equally

Table S1: Descriptive statistics stratified by age group and serological status

	**0-5 years**[1]			**6-11 years**[1]			**12-17 years**[1]		
	Negative N = 80	Positive N = 80	P-value	Negative N = 178[1]	Positive N = 267	P-value	Negative N = 206	Positive N = 223	P-value
**Sex**			0.2			0.2			0.7
Female	46 (57%)	36 (45%)		94 (53%)	123 (46%)		112 (54%)	117 (52%)	
Male	34 (42%)	44 (55%)		84 (47%)	143 (54%)		94 (46%)	106 (48%)	
Other	0 (0%)	0 (0%)		0 (0%)	1 (0%)		0 (0%)	0 (0%)	
**Chronic condition**[4]	9 (11%)	8 (10%)	0.8	38 (21%)	37 (14%)	0.039	73 (35%)	76 (34%)	0.8
**Confirmed SARS-CoV-2 infection**[5]	3 (4%)	16 (20%)**	0.001	12 (7%)	103 (39%)**	<0.001	10 (5%)	109 (49%)**	<0.001
**Confirmed SARS-CoV-2 <u>symptomatic</u> infection**	3 (4%)	14 (18%)**	0.005	9 (5%)	79 (30%)**	<0.001	9 (4%)	84 (38%)**	<0.001
**Vaccination Status**[7]						0.5			<0.001
No	47 (100%)	32 (100%)		116 (99%)	134 (100%)		48 (36%)	55 (48%)	
Yes, 1 dose				1 (1%)	0 (0%)		3 (2%)	27 (23%)	
Yes, 2 doses							83 (62%)	33 (29%)	
**Persistent symptoms**									
**Symptoms lasting over 4 weeks**	14 (18%)	11 (14%)	0.3	31 (17%)	36 (13%)	0.5	28 (14%)	52 (23%)*	0.01
Symptoms lasting 4 to 6 weeks	5 (6%)	4 (5%)	>0.9	15 (9%)	14 (5%)	0.2	7 (3%)	12 (5%)	0.3
Symptoms lasting 6 to 8 weeks	2 (2%)	0 (0%)	0.5	4 (2%)	4 (2%)	0.7	7 (3%)	10 (4%)	0.6
Symptoms lasting 8 to 12 weeks	4 (5%)	0 (0%)	0.12	2 (1%)	2 (1%)	>0.9	2 (1%)	2 (1%)	>0.9
Symptoms lasting over 12 weeks	3 (4%)	7 (9%)	0.2	10 (6%)	16 (6%)	0.9	12 (5%)	31 (14%)*	0.05

[1]Two-sided Fisher's exact test or Pearson's Chi-squared test,
*indicates p-value <0.05
**indicates p-value <0.01

Table S2: Characteristics of seropositive children with symptoms lasting over 12 weeks.

Characteristic	n = 54
**Sex**	
Female	25 (46%)
Male	29 (54%)
Other	0 (0%)
**Age group (years)**	
0-5	7 (13%)
6-11	16 (30%)
12-17	31 (57%)
**Confirmed SARS-CoV-2 infection[1]**	30 (56%)
**Confirmed SARS-CoV-2 symptomatic infection[1]**	26 (48%)
**Persistent symptoms declared after infection**	17/30 (57%)

[1]Diagnosed COVID-19 with a positive test (PCR, antigen test)

Table S3: Chronic condition among children who experienced symptoms lasting over 12 weeks

Characteristics	Overall, N = 40	Seronegative, n = 13	Seropositive, n = 27	Sex and age-adjusted difference[a] (Percent, 95%CI)	P-value
**Asthma**	10 (25%)	1 (8%)	9 (33%)	25.3% (2.7;48.5)*	0.02
**Migraine**	7 (18%)	2 (15%)	5 (19%)	4.0% (-21.0;27.6)	>0.9
**Obesity**	3 (8%)	1 (8%)	2 (7%)	0.3 % (-17.8;17.2)	>0.9
**Osteo-articular[b]**	5 (12%)	2 (15%)	3 (11%)	-4.0 % (27.2;18.6)	0.6
**Dermatological[c] condition**	19 (20%)	10 (31%)	9 (15%)	-16.0% (-44.4;12.4)	0.3

*indicates p-value <0.05 using a two-sided Likelihood Ratio Test, without adjustments for multiple comparisons.
[a]Adjusting for age and sex
[b]Disease or malformation of the skeleton, joints, or muscles (e.g. kypho-scoliosis, scoliosis, lordosis, hip dysplasia, tendon rupture without trauma)
[c]Eczema, psoriasis,etc.

Table S4: Descriptive statistics of symptoms lasting over 12 weeks stratified by serological status

	Overall<br>N = 79	Seronegative<br>N = 25	Seropositive<br>N = 54	P-value[1]
**General**				
Fever	2 (3%)	0 (0%)	2 (4%)	>0.9
Fatigue	19 (24%)	5 (20%)	14 (26%)	0.6
Trouble concentrating	11 (14%)	1 (4%)	10 (19%)	0.2
Less motivation	19 (24%)	7 (28%)	12 (22%)	0.6
Low mood	22 (28%)	8 (32%)	14 (26%)	0.6
Dizziness after physical effort	3 (4%)	1 (4%)	2 (4%)	>0.9
Malaise	2 (3%)	1 (4.0%)	1 (2%)	0.5
Nervousness	10 (13%)	2 (8%)	8 (15%)	0.5
Anxiety	22 (28%)	10 (40%)	12 (22%)	0.10
Hair loss	1 (1%)	1 (4%)	0 (0%)	0.3
**Respiratory**				
Cough	3 (4%)	0 (0%)	3 (6%)	0.5
Sore throat	5 (6%)	1 (4%)	4 (7%)	>0.9
Dripping nose	9 (11%)	1 (4%)	8 (15%)	0.3
Difficulty breathing	5 (6%)	1 (4%)	4 (7%)	>0.9
**Gastrointestinal**				
Loss of appetite	6 (8%)	1 (4%)	5 (9%)	0.7
Nausea	7 (9%)	1 (4%)	6 (11%)	0.4
Vomiting	3 (4%)	0 (0%)	3 (6%)	0.5
Constipation	4 (5%)	0 (0%)	4 (7%)*	0.05
Diarrhea	4 (5%)	1 (4%)	3 (6%)	>0.9
Weight gain	5 (6%)	2 (8%)	3 (6%)	0.6
Weight loss	6 (8%)	1 (4%)	5 (9%)	0.7
Abdominal pain	15 (19%)	1 (4%)	14 (26%)*	0.03
**Musculoskeletal**				
Muscle pain	8 (10%)	1 (4%)	7 (13%)	0.4
Joint pain	8 (10%)	2 (8%)	6 (11%)	>0.9
**Neurological**				
Headache	10 (13%)	2 (8%)	8 (15%)	0.5
Smell loss	7 (9%)	0 (0%)	7 (13%)*	0.04
Taste loss	1 (1%)	0 (0%)	1 (2%)	>0.9
Dizziness	4 (5%)	1 (4%)	3 (4%)	>0.9
Language problem	2 (3%)	0 (0%)	2 (4%)	0.5
Insomnia	12 (15%)	5 (20%)	7 (13%)	0.09
Hypersomnia	4 (5%)	1 (4%)	3 (6%)	>0.9
**Cardiovascular**				
Palpitations	3 (4%)	0 (0%)	3 (6%)	0.5
Chest pain	2 (3%)	1 (4%)	1 (2%)	0.5
**Genito-urinary**				
Urination problem	4 (5%)	2 (8%)	2 (4%)	0.6
Menstruation modification	1 (1%)	0 (0%)	1 (2%)	>0.9
**Dermatological**				
Skin rash	9 (11%)	3 (12%)	6 (11%)	0.6
Itching skin	6 (8%)	4 (16%)	2 (4%)	>0.9

[1]Two sided Fisher's exact test or Pearson's Chi-squared test, without adjustments for multiple comparisons

*indicates p-value <0.05

Questions asked to identify persistent symptoms

Q12. Please tick in the following list if since the start of the pandemic, the child has suffered from one or more new symptoms that lasted 4 consecutive weeks or more? (Several answers possibles)

☐ He or she had no new symptoms that lasted 4 weeks or more (skip to question 13)

General symptoms

☐ Fever, body temperature 37.5°C or higher
☐ Fatigue, exhaustion
☐ Trouble concentrating
☐ Loss of motivation
☐ Low morale
☐ Weakness
☐ Diziness after physical effort
☐ Nervousness, not holding still
☐ Anxiety
☐ Hair loss

Respiratory symptoms

☐ Persistent cough
☐ Sore throat
☐ Runny nose, blocked nose or itchy/burning nose
☐ Unusual shortness of breath on exertion

Gastrointestinal symptoms

☐ Loss of appetite
☐ Nausea
☐ Vomiting
☐ Constipation
☐ Diarrhea
☐ Weight gain
☐ Weight loss
☐ Stomach/abdominal pain

Musculoskeletal symptoms

☐ Muscle pain
☐ Joint pain

Neurological symptoms

☐ Headache
☐ Change or loss of smell
☐ Change or loss of taste
☐ Dizziness (e.g. when going from lying/sitting to standing or standing for a long time)
☐ Recurring fainting
☐ Vision problems / blurred vision
☐ Tremors
☐ Tingling/“pins and needles” sensation
☐ Damage to the extremities (e.g. fingers/toes becoming cold or warm, very pale or red)
☐ Language or communication problems
☐ Insomnia (difficulty falling asleep, difficulty staying asleep)
☐ Excessive daytime sleepiness or prolonged nocturnal sleep

Cardiovascular symptoms

☐ Palpitations (heartbeats felt to be very strong or disordered)
☐ Chest pain

Dermatological symptoms

☐ Urinary problems
☐ Changes in menstruation
☐ Dermatological symptoms
☐ Skin rash (pimples or redness on the skin)

☐ Itchy skin
☐ Redness, itching and/or discharge from the eyes

If at least one symptom is checked
Q12.1 When did the child first develop these symptoms?
………………………….

Q12.2 How long did these symptoms last or how long have they lasted if still present?
☐ 4 to 6 weeks
☐ More than 6 weeks to 8 weeks
☐ More than 8 weeks to 12 weeks
☐ More than 12 weeks

Q12.3 Considering the most severe symptom, to what extent did this symptom affect the child's daily life (on a scale of 1 very weak limitation - 10 strong limitation) …………………

Table S5: Sex and age- adjusted and un-adjusted prevalence and prevalence difference of persistent symptoms

**Age group (years)**	**Serological status**	**Prevalence of symptoms lasting over 12 weeks Percent (95%CI)**		**Prevalence difference of symptoms lasting over 12 weeks Percent (95%CI)**	
		Unadjusted	Adjusted for age and sex, when appropriate	Unadjusted	Adjusted for age and sex, when appropriate
Female (n=528)					
	Negative	5.5 (2.7,8.4)	4.9 (2.2;7.6)		
	Positive	9.1 (5.7,12.5)	8.3 (4.9;11.6)		
	**Difference**			3.5 (-1.2,8.3)	3.4 (-1.1;8.4)
Male (n=505)					
	Negative	5.2 (2.2,8.2)	5.0 (2.12;8.0)		
	Positive	9.9 (6.5,13.3)	9.7 (6.4;13.2)		
	**Difference**			4.7 (0.5,9.0)	4.7 (0.2;9.4)
0-5 (n=160)					
	Negative	3.8 (0.0,8.01)	3.8 (0.0;8.1)		
	Positive	8.7 (2.5,14.9)	8.0 (1.8;14.2)		
	**Difference**			4.9 (-3.6;13.5)	4.2 (-4.4;13.3)
6-11 (n=445)					
	Negative	5.6 (2.2;9.0)	5.3 (2.0;8.6)		
	Positive	6.0 (3.1;8.9)	5.3 (2.6;8.1)		
	**Difference**			0.44 (-4.3;5.2)	0.0 (-5.2;5.2)
12-17 (n=429)					
	Negative	5.8 (2.6;9.0)	5.3 (2.3;8.4)		
	Positive	13.9 (9.4;18.5)	13.6 (9.3;18.1)		
	**Difference**			8.1 (2.4;13.8)	8.3 (3.5;13.5)
All ages (N=1034)					
	Negative	5.4 (3.4;7.4)	5.0 (3.0;7.1)		
	Positive	9.5 (7.1;11.9)	9.1 (6.7;11.8)		
	**Difference**			4.1 (1.1;7.1)	4.1 (1.1;7.3)

[a]As presented in Table 1, one child had no biological sex assigned.

Table S6: Adjusted prevalence ratio of symptoms lasting over 12 weeks

**Report of symptoms lasting over 12 weeks**		**Overall[a] N=1034[b] PR (95%CI)**			**Within seropositives[a] n= 570[b] PR (95%CI)**			**Within seronegatives[a] n=464[b] PR (95%CI)**		
		**Unadjusted**	**Adjusted**	**p-value**	**Unadjusted**	**Adjusted**	**p-value**	**Unadjusted**	**Adjusted**	**p-value**
**Sex**	Female	1.0 (ref)	1.0 (ref)	-	1.0 (ref)	1.0 (ref)	-	1.0 (ref)	1.0 (ref)	
	Male	1.1 (0.7-1.6)	1.1 (0.8;1.6)	0.665	1.1 (0.7-1.7)	1.1 (0.7;1.8)	0.723	0.9 (0.4-1.9)	0.9 (0.4-1.9)	0.875
**Age (years)**		1.1 (1.0-1.2)*	1.1 (1.0;1.2)*	0.012	1.1 (1.0-1.2)*	1.1 (1.0;1.3)*	0.021	1.1 (0.9-1.1)	1.1 (0.9-1.2)	0.261
**Serological status**	Seronegative	1.0 (ref)	1.0 (ref)	-	-	-	-	-		-
	Seropositive	1.8 (1.1;2.8)**	1.8 (1.2;2.8)**	<0.01	-	-	-	-		-
**Chronic condition**	No	1.0 (ref)	1.0 (ref)	-	1.0 (ref)	1.0 (ref)	-	1.0 (ref)	1.0 (ref)	-
	Yes	3.4 (2.2-5.1)**	3.6 (2.3;5.5)**[c]	<0.01	3.6 (2.2-6.0)**	3.5 (2.0;6.1)**[c]	<0.01	3.1 (1.4-6.5)**[c]	2.9 (1.3-6.5)**[c]	<0.01
**Parental education**	Tertiary	1.0 (ref)	1.0 (ref)	-	1.0 (ref)	1.0 (ref)	-	1.0 (ref)	1.0 (ref)	-
	Secondary	1.4 (0.8-2.6)	1.2 (0.7;2.0)[c]	0.471	1.4 (0.8-2.5)	1.3 (0.6;2.5)[c]	0.469	1.4 (0.8;2.5)[c]	1.2 (0.7;2.0)[c]	0.469
	Primary	1.7 (0.5-5.4)	1.9 (0.8;4.7)[c]	0.365	1.7 (0.6-5.4)	1.7 (0.5;5.2)[c]	0.369	1.7 (0.6;5.3)[c]	1.9 (0.8;4.6)[c]	0.369
**Financial situation**	High	1.0 (ref)	1.0 (ref)	-	1.0 (ref)	1.0 (ref)	-	1.0 (ref)	1.0 (ref)	-
	Average to poor	3.4 (1.6-6.9)*	2.5 (1.4;4.6)*[c]	<0.05	3.4 (1.6-6.9)*	3.0 (1.5;6.2)*[c]	<0.05	1.3 (0.8-3.6)[c]	1.2 (0.9-3.4)[c]	0.501

[a]Prevalence ratio and 95% confidence interval are from Poisson regression with random effect on the household using the GLMMadaptive package in R, using a two-sided Likelihood Ratio Test, without adjustments for multiple comparisons.
[b]Complete case analysis. For each model presented, missing data in the covariates were excluded.
[c]Adjusting for age and sex
*indicates p-value <0.05
**indicates p-value <0.01
