# Supplementary material for: A population-based serological study of post-COVID syndrome prevalence and risk factors in children and adolescents
Source: Nat Commun. 2022 Nov 29;13:7086. doi: 10.1038/s41467-022-34616-8 (PMC9708639; doi:10.1038/s41467-022-34616-8)
Supplement: Supplementary file 3 — Reporting Summary [file 41467_2022_34616_MOESM3_ESM.pdf]

nature portfolio

Corresponding author(s): Pr. Idris Guessous

Last updated by author(s): Oct 25, 2022

Reporting Summary

Nature Portfolio wishes to improve the reproducibility of the work that we publish. This form provides structure for consistency and transparency in reporting. For further information on Nature Portfolio policies, see our Editorial Policies and the Editorial Policy Checklist.

Statistics

For all statistical analyses, confirm that the following items are present in the figure legend, table legend, main text, or Methods section.

n/a	Confirmed	
☐	☒	The exact sample size (n) for each experimental group/condition, given as a discrete number and unit of measurement
☐	☒	A statement on whether measurements were taken from distinct samples or whether the same sample was measured repeatedly
☐	☒	The statistical test(s) used AND whether they are one- or two-sided<br>*Only common tests should be described solely by name; describe more complex techniques in the Methods section.*
☐	☒	A description of all covariates tested
☐	☒	A description of any assumptions or corrections, such as tests of normality and adjustment for multiple comparisons
☐	☒	A full description of the statistical parameters including central tendency (e.g. means) or other basic estimates (e.g. regression coefficient) AND variation (e.g. standard deviation) or associated estimates of uncertainty (e.g. confidence intervals)
☐	☒	For null hypothesis testing, the test statistic (e.g. F, t, r) with confidence intervals, effect sizes, degrees of freedom and P value noted<br>*Give P values as exact values whenever suitable.*
☒	☐	For Bayesian analysis, information on the choice of priors and Markov chain Monte Carlo settings
☒	☐	For hierarchical and complex designs, identification of the appropriate level for tests and full reporting of outcomes
☒	☐	Estimates of effect sizes (e.g. Cohen's d, Pearson's r), indicating how they were calculated

Our web collection on statistics for biologists contains articles on many of the points above.

Software and code

Policy information about availability of computer code

Data collection	The data were collected using the softwares Formstack® (02.2022) and sugarCRM® (version 4.2).
Data analysis	All analyses were performed with R (version 4.0.3), using GLMMadaptive (0.8-5), dplyr (1.0.10), gtsummary (1.6.2) packages. Our computer code are accessible to researchers upon request to the corresponding author.

For manuscripts utilizing custom algorithms or software that are central to the research but not yet described in published literature, software must be made available to editors and reviewers. We strongly encourage code deposition in a community repository (e.g. GitHub). See the Nature Portfolio guidelines for submitting code & software for further information.

Data

Policy information about availability of data

All manuscripts must include a data availability statement. This statement should provide the following information, where applicable:

- Accession codes, unique identifiers, or web links for publicly available datasets
- A description of any restrictions on data availability
- For clinical datasets or third party data, please ensure that the statement adheres to our policy

Participants' informed consent did not authorize data to be immediately publicly available. It does allow, however, for the data to be made available to the scientific community upon submission of a data request application to the investigators board via the corresponding author.

Human research participants

Policy information about studies involving human research participants and Sex and Gender in Research.

Reporting on sex and gender	The term sex (biological attribute) was used in this study and reported by parents in a questionnaire related to their child(ren)
Population characteristics	Our sample was collected from December 2021 to February 2022 and included 1034 children aged 6 months to 17 years from 612 households: 505 (49%) were girls and the mean age was 10.2 years [SD=4.2]). Overall, 785 (76%) children had parents with a tertiary education, 200 (19%) with a secondary education and 42 (4%) with a primary education level (7 missing data). Among our participants, 150 (15%) were reported to live in a household with an average to poor financial situation; and 270 (26%) were reported to have a chronic medical condition. There were 570 (55%) children who tested positive for anti-SARS-CoV-2 N antibodies.
Recruitment	Participants were invited to participate to our study by postal letter or by email when available.<br>Individuals with favourable socio-economic conditions were more likely to participate. This could have led to an underestimation of the prevalence of post-COVID since their occurrence was more common among underprivileged individuals. Overall, this might limit the representativeness of our results.
Ethics oversight	The study was approved by the Cantonal Research Ethics Commission of Geneva, Switzerland (ID 2021-01973).

Note that full information on the approval of the study protocol must also be provided in the manuscript.

Field-specific reporting

Please select the one below that is the best fit for your research. If you are not sure, read the appropriate sections before making your selection.

☐ Life sciences ☒ Behavioural & social sciences ☐ Ecological, evolutionary & environmental sciences

For a reference copy of the document with all sections, see nature.com/documents/nr-reporting-summary-flat.pdf

Behavioural & social sciences study design

All studies must disclose on these points even when the disclosure is negative.

Study description	The study is quantitative, observational and cross-sectional.
Research sample	Eligibility criteria for the SEROCoV-KIDS study included being between 6 months and 17 years old and residing in the Canton of Geneva at the time of enrolment. In the present study, we analysed 1034 children and adolescents recruited between December 1st, 2021 and February 16th, 2022. 505 (49%) were girls and the mean age was 10.2 years [SD=4.2]). Overall, 785 (76%) children had parents with a tertiary education level, 200 (19%) with a secondary and 42 (4%) with a primary education level (7 missing data), while 150 (15%) lived in a household with an average to poor financial situation. Therefore, individuals with favourable socio-economic conditions were more likely to participate, which is often the case in this type of study. This might limit the representativeness of our results.
Sampling strategy	Children and adolescents in the SEROCoV-KIDS cohort were either newly selected from random samples obtained from lists of state registries or had a household member already participating in a population-based COVID-19 seroprevalence study conducted by our group. They were all invited to participate following a standardised procedure, 20% accepted to participate.
Data collection	At the baseline assessment, all children and adolescents were invited to perform a serological test (by blood drawing) to measure anti-SARS-CoV-2 antibodies (anti-N). One of the parent or legal guardian (referent parent) was asked to fill out online questionnaires related to health and development for him/herself and for each of his/her children, on the Specchio-COVID19 secured digital platform. No researcher was present when the parents answered the questionnaire. This is an observational study, so blinding is not applicable.
Timing	We selected all children who were included from December 1st, 2021 until February 16th, 2022
Data exclusions	Participants with missing data in at least one of the covariates were excluded from the models (9 were removed).
Non-participation	Among invited 3060 households, 625 households participated in our study (participation rate of 20.4%). Reasons of non-participation were not recorded.
Randomization	This is an observational study, randomization is not applicable.

Reporting for specific materials, systems and methods

We require information from authors about some types of materials, experimental systems and methods used in many studies. Here, indicate whether each material, system or method listed is relevant to your study. If you are not sure if a list item applies to your research, read the appropriate section before selecting a response.

Materials & experimental systems

n/a	Involved in the study
☐	☒ Antibodies
☒	☐ Eukaryotic cell lines
☒	☐ Palaeontology and archaeology
☒	☐ Animals and other organisms
☒	☐ Clinical data
☒	☐ Dual use research of concern

Methods

n/a	Involved in the study
☒	☐ ChIP-seq
☒	☐ Flow cytometry
☒	☐ MRI-based neuroimaging

Antibodies

Antibodies used	Serological tests were based on the semiquantitative commercially-available immunoassay Roche Elecsys anti-SARS-CoV-2 N, detecting total Ig (including IgG) against the nucleocapsid protein of the SARS-CoV-2 virus
Validation	The test has an in-house sensitivity of 99.8% (95% CrI, 99.4%-100%) and specificity of 99.1% (95% CI, 98.3%-99.7%) (Roche Diagnostics, Rotkreuz, Switzerland).
